# Arterial baroreflex control of muscle sympathetic nerve activity under orthostatic stress in humans

**DOI:** 10.3389/fphys.2012.00314

**Published:** 2012-08-07

**Authors:** Masashi Ichinose, Takeshi Nishiyasu

**Affiliations:** ^1^Human Integrative Physiology Laboratory, School of Business Administration, Meiji UniversityTokyo, Japan; ^2^Institute of Health and Sport Sciences, University of TsukubaIbaraki, Japan

**Keywords:** sympathetic nervous system, lower body negative pressure, integrated circulatory regulation, blood pressure, peripheral reflexes

## Abstract

The mechanisms by which blood pressure is maintained against the orthostatic stress caused by gravity's effect on the fluid distribution within the body are important issues in physiology, especially in humans who usually adopt an upright posture. Peripheral vasoconstriction and increased heart rate (HR) are major cardiovascular adjustments to orthostatic stress and comprise part of the reflex response elicited via the carotid sinus and aortic baroreceptors (arterial baroreflex: ABR) and cardiopulmonary stretch receptors (cardiopulmonary baroreflex). In a series of studies, we have been characterizing the ABR-mediated regulation of cardiovascular hemodynamics and muscle sympathetic nerve activity (MSNA) while applying orthostatic stress in humans. We have found that under orthostatic stress, dynamic carotid baroreflex responses are modulated as exemplified by the increases in the MSNA, blood pressure, and HR responses elicited by carotid baroreflex unloading and the shorter period of MSNA suppression, comparable reduction and faster recovery of mean arterial blood pressure (MAP) and greater HR response to carotid baroreflex stimulation. Our results also show that ABR-mediated beat-to-beat control over burst incidence, burst strength and total MSNA is progressively modulated as orthostatic stress is increased until induction of syncope, and that the sensitivity of ABR control over the aforementioned MSNA variables is substantially reduced during the development of syncope. We suggest that in humans, the modulation of ABR function under orthostatic stress may be one of the mechanisms by which blood pressure is maintained and orthostatic hypotension limited, and impairment of ABR control over sympathetic vasomotor activity leads to the severe hypotension associated with orthostatic syncope.

## Introduction

The mechanisms involved in blood pressure regulation under conditions of orthostatic stress are an important research issue in physiology, especially with respect to humans, who usually adopt an upright posture (Rowell, 1986). With the body in an upright posture, blood in the central circulation pools in the peripheral veins, thus reducing the cardiac filling pressure and leading to a reduction in arterial blood pressure. Increases in peripheral vascular resistance and heart rate (HR), two major autonomic nervous system-mediated cardiovascular adjustments to orthostatic stress (Rowell, 1986), form part of the reflex response pattern elicited via the carotid sinus and aortic baroreceptors (arterial baroreflex: ABR) and cardiopulmonary stretch receptors (cardiopulmonary baroreflex) (Zoller et al., 1972; Johnson et al., 1974; Sundlof and Wallin, 1978a; Mancia and Mark, 1983; Mark and Mancia, 1983; Rowell, 1986; Pawelczyk and Raven, 1989; Eckberg and Sleight, 1992; Nishiyasu et al., 1993, 1999; Taylor et al., 1995). Peripheral vascular resistance and muscle sympathetic nerve activity (MSNA) both increase progressively as the severity of orthostatic stress increases in humans (Johnson et al., 1974; Sundlof and Wallin, 1978a; Rowell, 1986; Victor and Leimbach, 1987; Pawelczyk and Raven, 1989; Vissing et al., 1989; Nishiyasu et al., 1993; Khan et al., 2002; Convertino et al., 2004; Ichinose et al., 2004a,b), and it has been assumed that the level of sympathetic vasomotor activity elicited in response to orthostatic stress reflects the degree of unloading of the arterial and cardiopulmonary baroreceptors (Zoller et al., 1972; Johnson et al., 1974; Sundlof and Wallin, 1978a; Rowell, 1986; Eckberg and Sleight, 1992; Taylor et al., 1995).

The hypothesis that ABR-mediated cardiovascular control is modulated by orthostatic stress (Bevegård et al., 1977; Ebert, 1983; Mancia and Mark, 1983; Mark and Mancia, 1983; Victor and Mark, 1985; Pawelczyk and Raven, 1989; Shi et al., 1993, 1997) is supported by extensive evidence from both animal (Koike et al., 1975; Mancia and Mark, 1983; Mark and Mancia, 1983) and human studies (Bevegård et al., 1977; Ebert, 1983; Victor and Mark, 1985; Pawelczyk and Raven, 1989; Shi et al., 1993, 1997; Ogoh et al., 2002). In humans, for example, Victor and Mark (1985) showed that forearm vasoconstriction induced by neck pressure (NP; i.e., carotid baroreceptor unloading) is greatly augmented by application of lower body negative pressure (LBNP; −10 mmHg), and that the augmented response greatly exceeds the sum of the individual reflex responses (i.e., the response to LBNP alone plus the response to NP alone). In addition, Pawelczyk and Raven (1989) demonstrated that the maximum gain of the carotid baroreflex control of blood pressure and R-R interval (as calculated from the entire carotid baroreflex stimulus-response curve) increases progressively with decreases in central venous pressure induced by increasing the LBNP level (0 to −50 mmHg LBNP). Ogoh et al. (2002) reported that although the sensitivity of the carotid baroreflex control of HR and mean arterial blood pressure (MAP) were unaffected, the contribution made by the evoked change in total vascular conductance to the peak MAP response to a 5 s NP or suction stimulus was greater when the subjects were in an upright, seated position than when they were supine. Enhancement of ABR control under orthostatic stress ought to be an excellent defense for an organism against orthostatic hypotension; however, the modulation of ABR function during orthostatic stress and its consequences are not fully understood. In this review we will focus our discussion on the modulation of ABR-mediated cardiovascular regulation in human subjects experiencing orthostatic stress in recently conducted studies of (1) ABR dynamic responses and (2) ABR-mediated beat-to-beat control of MSNA.

## Modulation of the ABR dynamic response under mild orthostatic stress

To test the hypothesis that ABR dynamic responses are modulated under mild orthostatic stress, we investigated the time course of carotid baroreflex-induced alterations in MSNA, MAP, and HR in the control situation and during LBNP (−15 mmHg) (Ichinose et al., 2004a). Carotid baroreceptor activity changed rapidly in response to 5 s of either NP (50 mmHg pressure) or neck suction (NS; 60 mmHg suction) applied using a neck chamber. To minimize respiratory-related modulation of HR, MAP, and MSNA, all neck-chamber stimuli were delivered while the subject held his/her breath. The total duration of the voluntary apnea was 13 s (a 3 s pre-stimulus period, a 5 s stimulus, and a 5 s post-stimulus period). To assess the effect of the apnea itself, measurements were repeated during breath-holding, but with neck-chamber pressure was maintained at the ambient pressure. In each subject, four episodes each of NP and NS, and four episodes of apnea alone were applied in the control situation and again during LBNP. The 13 s records of the MSNA, MAP, and HR collected during the four sets of NP-NS trials were averaged and integrated over 1 s periods to allow us to assess the time course of MSNA, MAP, and HR responses. For this analysis, the mean voltage neurogram was advanced 1.3 s to allow for the conduction delay between the spinal cord and the recording site (Delius et al., 1972; Fagius and Wallin, 1980; Eckberg and Sleight, 1992). MSNA was found to increase slightly during breath-holding alone. To compensate for this effect, the MSNA responses recorded over the course of the four periods of breath-holding alone were averaged and the value so obtained was subtracted from the MSNA levels recorded before, during and after each neck-chamber stimulus. Hence, the MSNA responses to the neck-chamber stimuli are expressed as the change from the MSNA level recorded during breath-holding alone. The averaged MAP during the 3 s pre-stimulus period and the HR measured 1 s before the stimulus were taken as baseline values. Because breath-holding alone did not cause a significant change in MAP or HR from these baseline values, the responses of each are expressed as the absolute difference from its baseline value.

**Figure 1:**
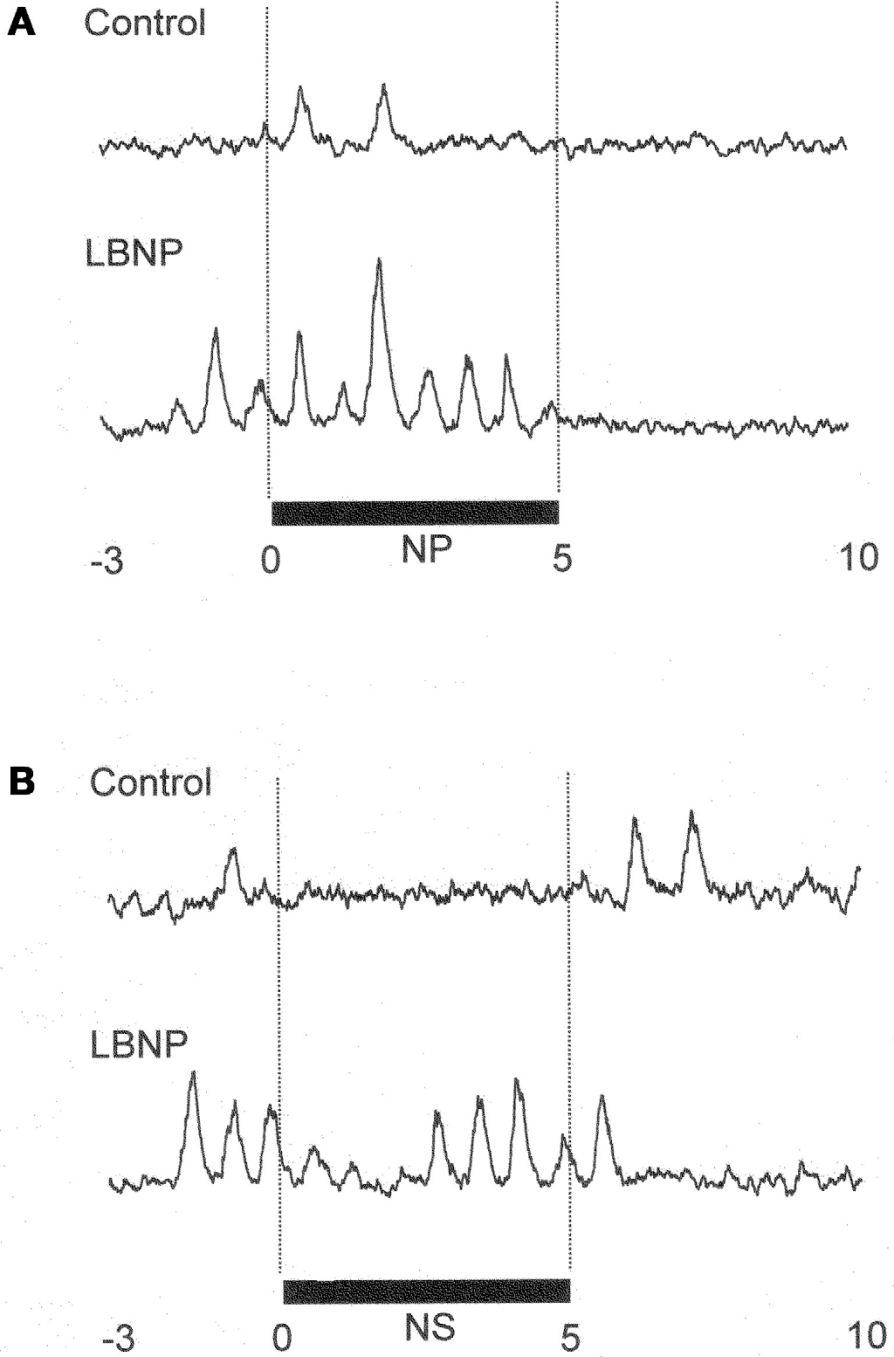
**MSNA response in one subject to application of +50 mmHg neck pressure (A) and −60 mmHg neck suction (B) in the control and LBNP situations**. NP, neck pressure; NS, neck suction. (Reproduced with permission from Ichinose et al., 2004a).

**Figure 2:**
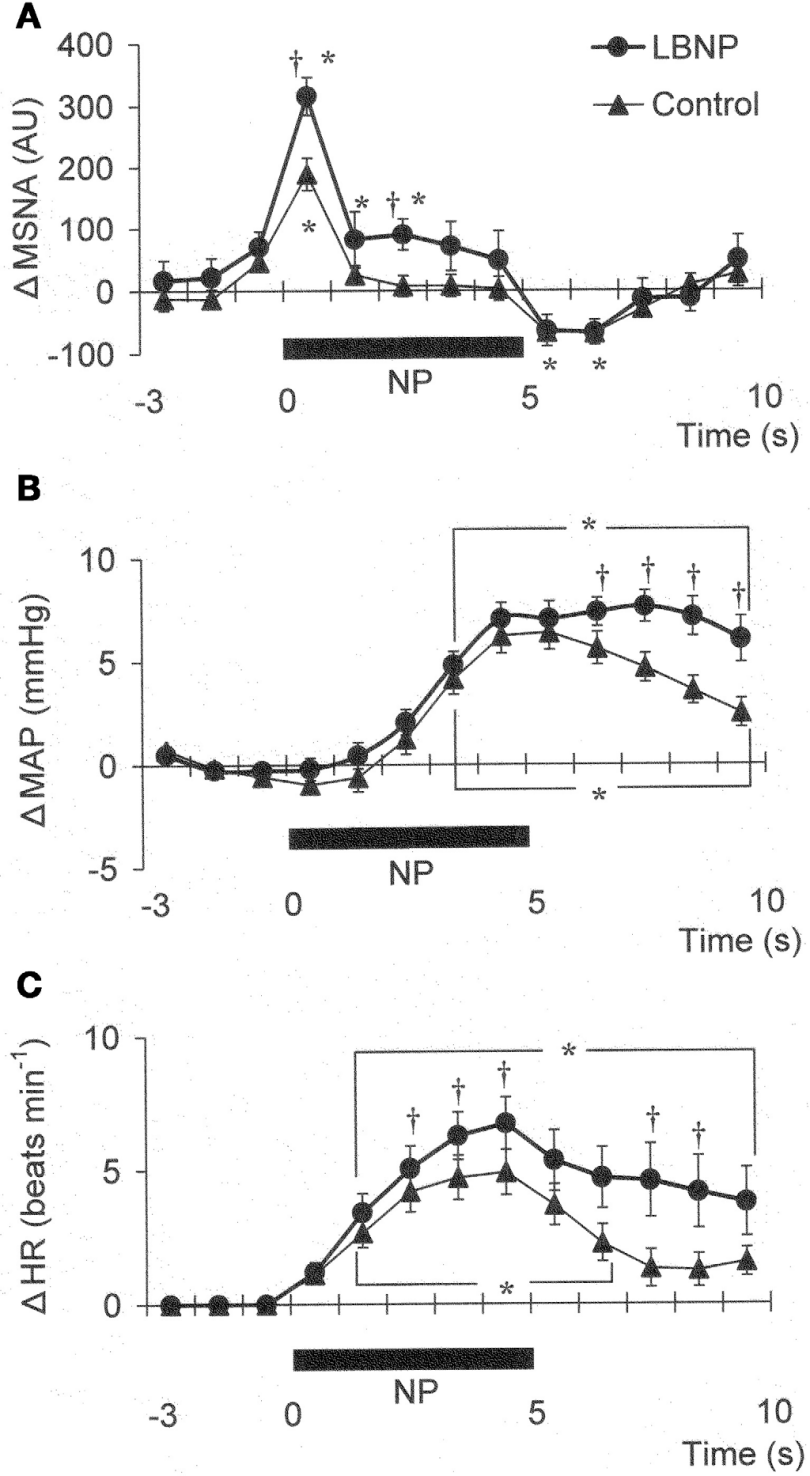
**Averaged reflex alterations of MSNA (A) (*n* = 10), MAP (B) (*n* = 12), and HR (C) (*n* = 12) elicited by neck pressure in the control and LBNP situations**. ^*^P < 0.05 vs. the value obtained 3 s prior to application of neck pressure. ^†^*P* < 0.05 vs. control. (Reproduced with permission from Ichinose et al., 2004a).

**Figure 3:**
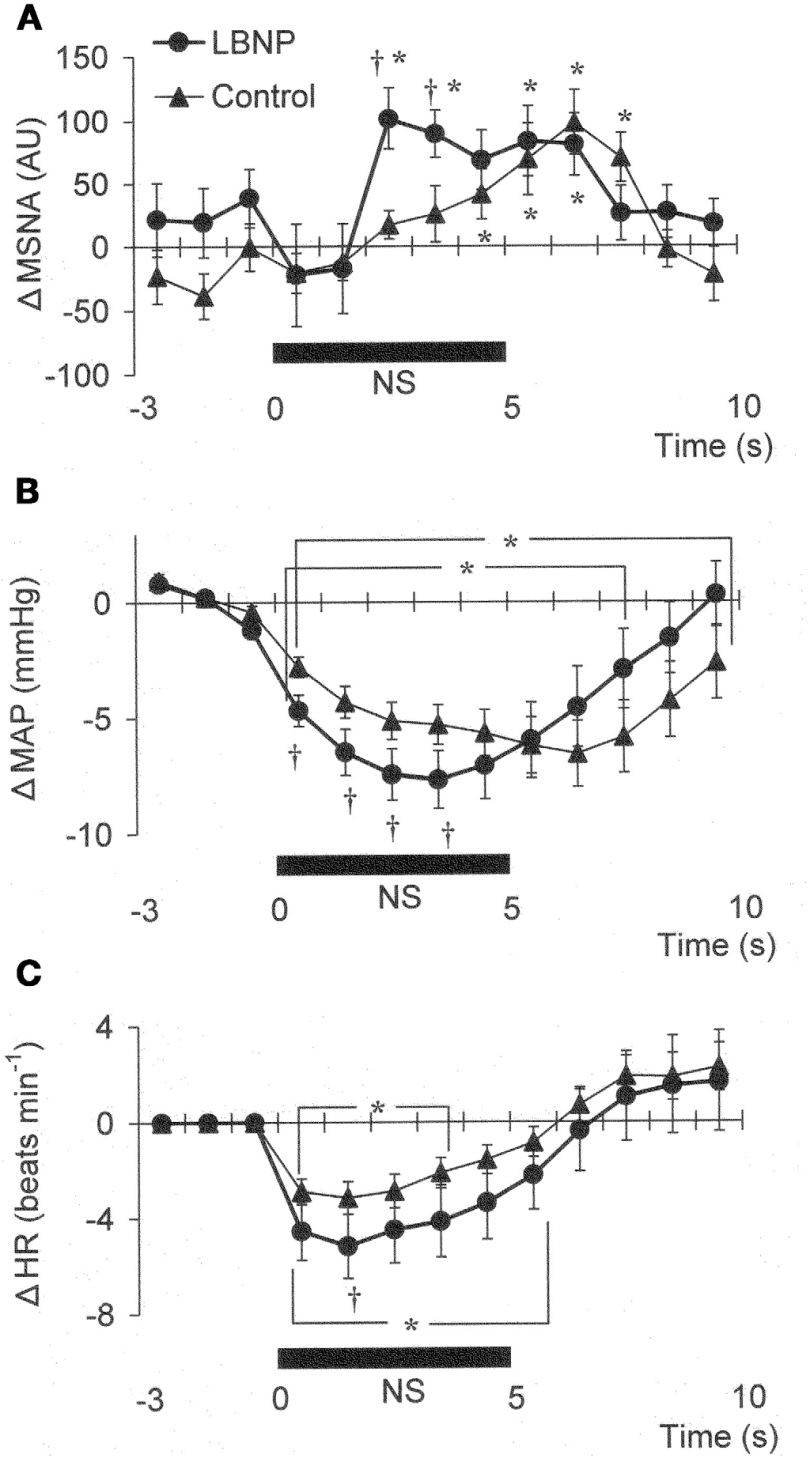
**Averaged reflex alterations in MSNA (A) (*n* = 10), MAP (B) (*n* = 12), and HR (C) (*n* = 12) elicited by neck suction in the control and LBNP situations**. ^*^P < 0.05 vs. the value obtained 3 s prior to application of neck pressure. ^†^*P* < 0.05 vs. control. (Reproduced with permission from Ichinose et al., 2004a).

Figure 1 shows the original recorded MSNA responses elicited in one subject by application of NP (A) and NS (B) in the control situation and during LBNP. The time courses of the MSNA, MAP, and HR responses to NP and NS are illustrated in Figures 2 and 3, respectively. In both situations, increments in MSNA evoked by NP were very transient, with a decay occurring even while the NP remained elevated. Moreover, there was an undershoot after the NP was returned to the ambient level. At both 1 and 3 s into the response to NP, MSNA was significantly greater during LBNP than control (Figure 2A). And because the MSNA data were advanced by 1.3 s to allow for conduction delays, the timing of the enhanced MSNA response was consistent with a reasonable baroreflex latency, and can therefore be taken to represent a carotid baroreflex-mediated effect. At 7 to 10 s after the start of NP (at or after the time at which the peak MAP response occurred), MAP remained significantly higher during LBNP than control. The delay between the sympathetic bursting and the rise in MAP could reflect the relatively slow process of neural activation of vascular smooth muscle contraction (Hirst and Edwards, 1989). Examination of the time course of the HR responses to NP (Figure 2C) revealed that the peak HR response occurred within 5 s after the start of NP in both the control and LBNP situations and that the HR response was greater during LBNP than control.

In Figure 1B, it can be seen that in the control situation, MSNA was depressed for nearly the entire 5 s period of NS, but that during LBNP the depression of MSNA was transient, with recovery beginning while the NS stimulus was still ongoing. Examination of the time course of the MSNA responses to NS (Figure 3A) revealed that in both the control situation and during LBNP, MSNA tended to be reduced (relative to the level immediately before NS) for the first 2 s of the NS stimulation. Unfortunately, the mean group data in Figure 3A do not show significant suppression during the first 2 s or so of the NS period (although suppression is clear in Figure 2). This is at least partly due to differences in the timing of MSNA bursts relative to the start of the NS period among the individual tests. Thereafter, the time courses of the changes in MSNA differed between the control and LBNP situations, with the pattern of change during LBNP shifted leftward (i.e., occurring earlier). Indeed, MSNA was significantly greater during LBNP than control 3 and 4 s after the start of NS (Figure 3A). This leftward shift in the time course of the MSNA elevation implies that the duration of any MSNA suppression induced by NS may be shortened during LBNP (Figure 3B). During the first 4 s of the NS period, the reduction in MAP was greater during LBNP than control, and after 9 s of NS, MAP had already recovered to its baseline level during LBNP but remained significantly lower than baseline in the control situation. In addition, the peak MAP response to NS was observed 2–4 s earlier during LBNP than control. Figure 3C, which shows the time course of the HR response to NS, reveals that the reduction in HR was greater during LBNP than control.

Modulation of carotid baroreflex function at levels of LBNP insufficient to cause hypotension (less than −20 mmHg) has been reported before; indeed, such levels of LBNP are presumed to unload cardiopulmonary baroreceptors with little or no effect on arterial baroreceptor afferent activity (Zoller et al., 1972; Johnson et al., 1974; Rowell, 1986). Some reports have concluded that the augmented response to carotid distension or compression seen during LBNP is evidence of the tonic inhibitory influence of the cardiopulmonary baroreflex over carotid baroreflex-mediated cardiovascular adjustments (Ebert, 1983; Victor and Mark, 1985; Pawelczyk and Raven, 1989). It should be noted, however, that the small reduction in central blood volume induced by mild LBNP is reportedly sufficient to reduce the dimensions of the aortic baroreceptive areas (Taylor et al., 1995). If this is so, it is difficult to conclude that the dynamic response induced by mild LBNP results solely from deactivation (unloading) of the cardiopulmonary baroreceptors. Nevertheless, on the basis of the results summarized above, we can at least say that in a situation in which mild orthostatic stress is associated with a simultaneous unloading of the cardiopulmonary and arterial baroreceptors, the dynamic alterations in MSNA induced by the carotid baroreflex will be modulated.

### Alteration of carotid baroreflex-mediated sympathetic vascular regulation under orthostatic stress

We found that MSNA, MAP, and HR responses to NP were all augmented during LBNP. The greater MSNA response would be expected to lead, via greater vasoconstriction, to a greater MAP response during LBNP (Figures 2A,B). In addition, any HR change induced by NP could alter cardiac output and thus affect the blood-pressure responses (Raven et al., 1997), so the increased HR response to NP seen during LBNP could have contributed to the augmented MAP response. However, the peak blood-pressure response was delayed relative to the peak HR response, particularly in the LBNP situation (Figures 2B,C). Moreover, Ogoh et al. (2002) showed that the peak MAP response, normally observed 6–8 s after the start of a 5 s neck stimulus, is mainly due to reflex changes in total vascular conductance, with changes in cardiac output having little effect. Taken together, the summarized results suggest the augmented MAP response to NP seen during LBNP reflects an augmentation of carotid baroreflex-induced vasoconstriction. These results therefore suggest that during mild orthostatic stress, there is an augmentation of the carotid baroreflex regulation of blood pressure during NP and that this occurs primarily via enhancement of the baroreflex effect on sympathetic nerve activity.

The present results show that mild orthostatic stress affects the time course (latency) of the MSNA and MAP responses to carotid baroreflex loading (Figures 3A,B). The initial, gradual reduction in MAP that occurred during NS was greater during LBNP than control. This early reduction in MAP is reportedly due to a reduction in cardiac output secondary to a change in HR (Raven et al., 1997; Ogoh et al., 2002). If so, the greater HR response to NS seen during LBNP may lead to a greater initial reduction in MAP (Figure 3C). In addition, the briefer MSNA suppression and earlier MSNA elevation during LBNP (Figure 3A) would have counteracted any vasodilator response that could have led to a decrement in peak MAP 6–8 s after the start of NS in the control situation. Consequently, the peak MAP response could occur earlier during LBNP. Perhaps the initial decline in systemic blood pressure visible in Figure 3B caused a rapid reflex reversal of NS-induced suppression of MSNA via extra-carotid (i.e., aortic) baroreceptors (Mancia and Mark, 1983; Sanders et al., 1989; Kawada et al., 2000). In the control situation, NS tended to suppress MSNA despite the falling MAP, which might be expected to exert an MSNA-augmenting effect. During LBNP, any tendency toward NS-induced MSNA suppression lasted for only the first 2 s, and the MSNA-suppressing effect was quickly diminished and overcome by an MSNA-augmenting effect, which might have been strengthened by the unloading of the cardiopulmonary baroreceptors. This result is consistent with our finding that the peak MSNA response to NP was increased during LBNP (Figure 2A). It is also noteworthy that during LBNP, the greater initial decline in systemic blood pressure seen during NS may enhance the MSNA augmentation. Be that as it may, our results indicate that during mild orthostatic stress, the carotid baroreflex is still capable of suppressing MSNA and reducing blood pressure in response to a hypertensive stimulus but that any MSNA suppression induced by the carotid baroreflex may be overcome sooner, resulting in a more rapid recovery in blood pressure.

It has been suggested that the afferent signal from the cardiopulmonary baroreceptors inhibits the ABR at site(s) within the central nervous system (CNS) (Victor and Mark, 1985; Pawelczyk and Raven, 1989; Shi et al., 1993, 1997; Ogoh et al., 2002, 2003). Given that the nucleus tractus solitarius (NTS) receives several viscerosensory inputs, including afferent signals from both the arterial and cardiopulmonary baroreceptors, interaction between baroreflexes may well take place within the NTS (Spyer, 1990; Jianhua et al., 1998; Potts et al., 2003). In particular, modulation of the time course of carotid baroreflex-mediated vascular sympathetic responses due to unloading of the cardiopulmonary baroreceptors, as described above, could be effected within such a site of integration. Still, the precise mechanism remains unknown.

### Alteration of carotid baroreflex-mediated HR control under orthostatic stress

We observed that the peak HR response to both NP and NS was increased during LBNP (−15 mmHg), with no change in the general time course of the response (Figures 2C, 3C). This observation is at variance with the earlier findings that HR is unaffected by unilateral carotid baroreflex unloading or loading during mild LBNP (Takeshita et al., 1979; Victor and Mark, 1985), but is consistent with the finding that the maximum gain of the carotid baroreflex control of the R-R interval is increased by mild LBNP (<20 mmHg) (Pawelczyk and Raven, 1989). It has also been shown that the HR responses to neck-chamber stimuli are predominantly mediated by carotid baroreflex control of cardiac parasympathetic activity (Eckberg, 1980). The arterial baroreceptor projections to the NTS are relayed to the nucleus ambiguus and modulate the activity of preganglionic parasympathetic motor neurons (Spyer, 1990), which means that if afferent inputs from arterial and cardiopulmonary baroreceptors do indeed interact within the NTS, it is there that the carotid baroreflex regulation of cardiac parasympathetic tone may be modulated. In addition, although HR did not change during mild LBNP in the aforementioned study, cardiac parasympathetic tone reportedly declines during mild LBNP (Taylor et al., 1995). Consequently, the change in cardiac parasympathetic activity induced by an abrupt change in the afferent input from the carotid baroreceptors may differ between control and LBNP. These results at least suggest that under conditions involving mild orthostatic stress, the carotid baroreflex control of HR, which is predominantly mediated through regulation of cardiac parasympathetic tone, would be modulated alongside the regulation of vascular sympathetic activity.

## Modulation of ABR-mediated beat-to-beat control of MSNA under orthostatic stress

The ABR is known to influence the incidence and strength of MSNA bursts on a beat-to-beat basis and is thought to be the major modulator of MSNA in humans (Delius et al., 1972; Wallin et al., 1975; Sundlof and Wallin, 1978a,b; Wallin and Eckberg, 1982; Fagius et al., 1985; Macefield and Wallin, 1999; Kienbaum et al., 2001). Some reports have suggested that there is differential control over the occurrence and strength of sympathetic bursts (Hjemdahl et al., 1989; Malpas and Ninomiya, 1992; Kienbaum et al., 2001). And although ABR-mediated cardiovascular control appears to be modulated by orthostatic stress (Victor and Mark, 1985; Pawelczyk and Raven, 1989; Eckberg and Sleight, 1992), it remains unclear whether and how beat-to-beat control of the occurrence and strength of MSNA bursts is modulated under orthostatic stress. Moreover, whether the change in the ABR control of overall MSNA under orthostatic stress can be explained by modulation of the baroreflex control of those two MSNA parameters has never been examined. According to Ogoh et al. (2004), carotid baroreflex control over HR and MAP is largely absent during head-up tilt-induced syncope. Although there are several conceivable explanations for the impairment of the baroreflex during the development of syncope (e.g., sympathoinhibition at orthostatic syncope and/or the baroreceptor stimulus is outside the functional range), the precise mechanism remains unclear. That said, the results of the study by Ogoh et al. (2004) raise the possibility that the sensitivity of ABR control over beat-to-beat MSNA is impaired during the development of orthostatic syncope, though this has never been tested. To investigate that issue, we devised a technique to use the linear relationship between spontaneous variations in diastolic arterial pressure (DAP) and MSNA (Ichinose et al., 2004b,c) to assess ABR control of MSNA based three parameters: burst incidence, burst strength, and total MSNA. Using this technique, ABR control of sympathetic nerve activity could be evaluated in a way that enabled ABR control of burst occurrence and burst strength to be considered separately. Moreover, if the ABR control of overall MSNA (i.e., total MSNA) were modulated, this analysis technique would enable us to examine the underlying relationship between the modification of the control of burst occurrence and strength and the modification of the control of total MSNA.

**Figure 4:**
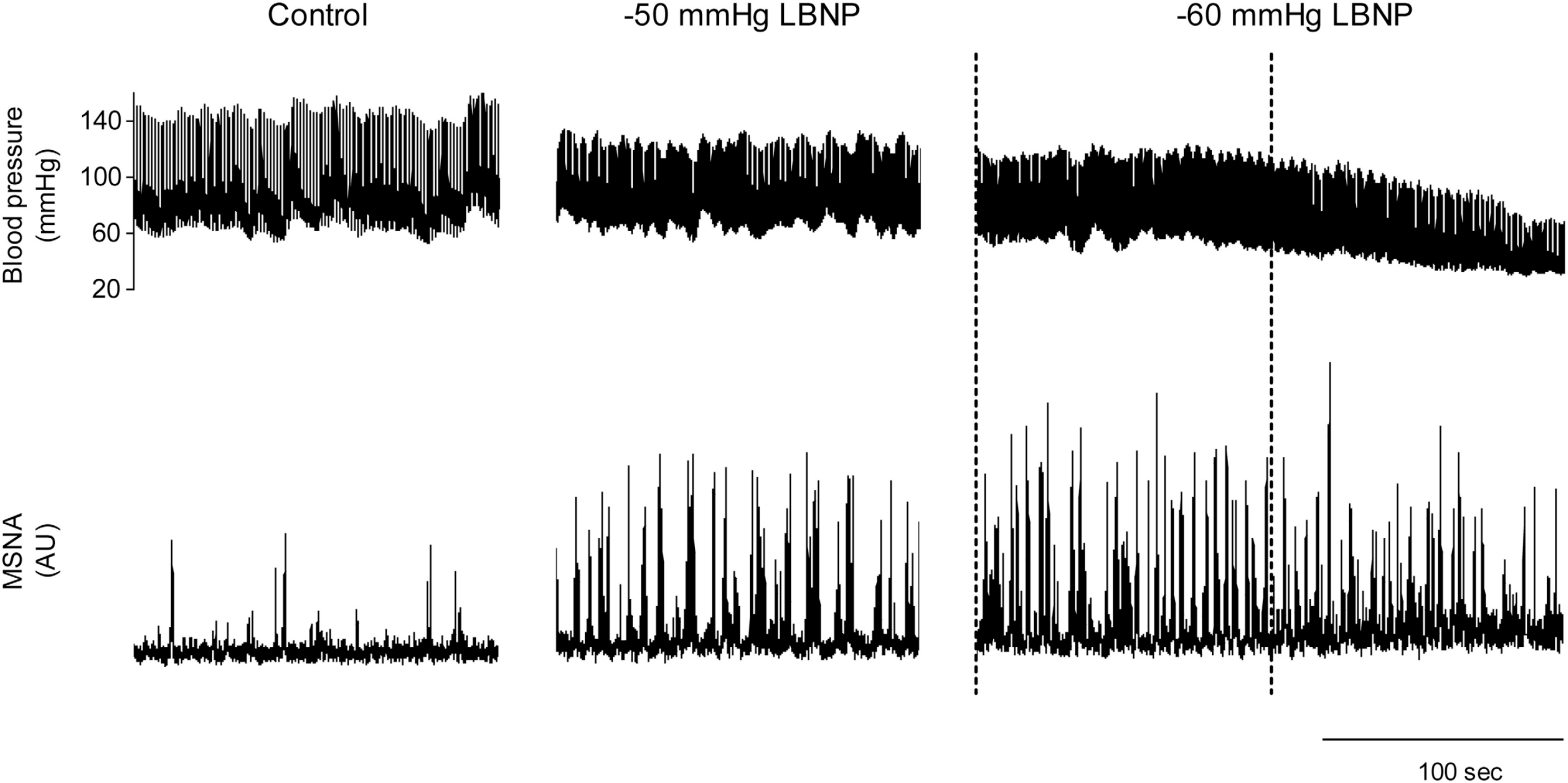
**Raw recordings of arterial blood pressure and MSNA in the control situation and at LBNPs of −50 and −60 mmHg in a subject who experienced symptoms of presyncope at −60 mmHg**. At LBNP = −60 mmHg, note the rapid decline in blood pressure and concomitant reduction in MSNA that occurred before the onset of syncope (the last part of the trace). With this subject, analysis of arterial baroreflex control over MSNA at LBNP = −60 mmHg was performed using the data obtained during the period between the dashed lines, when blood pressure and MSNA were largely stable. (Reproduced with permission from Ichinose et al., 2006).

**Figure 5:**
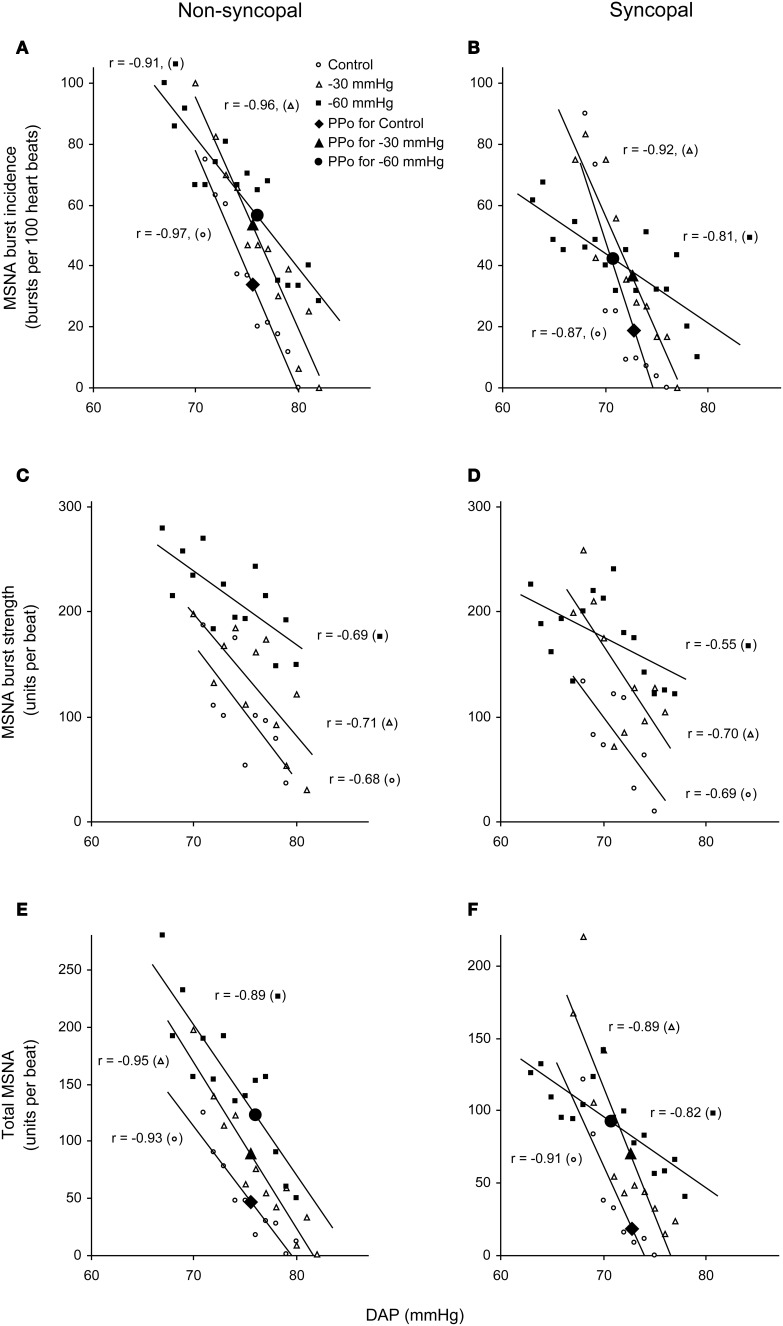
**Linear relationships between DAP and burst incidence (A,B), burst strength (C,D), and total MSNA (E,F) in the control situation and at LBNPs of −30 and −60 mmHg in a non-syncopal subject (A,C,E) and in a syncopal subject who experienced syncope at LBNP = −60 mmHg (B,D,F)**. PPo, prevailing point. (Reproduced with permission from Ichinose et al., 2006).

**Figure 6:**
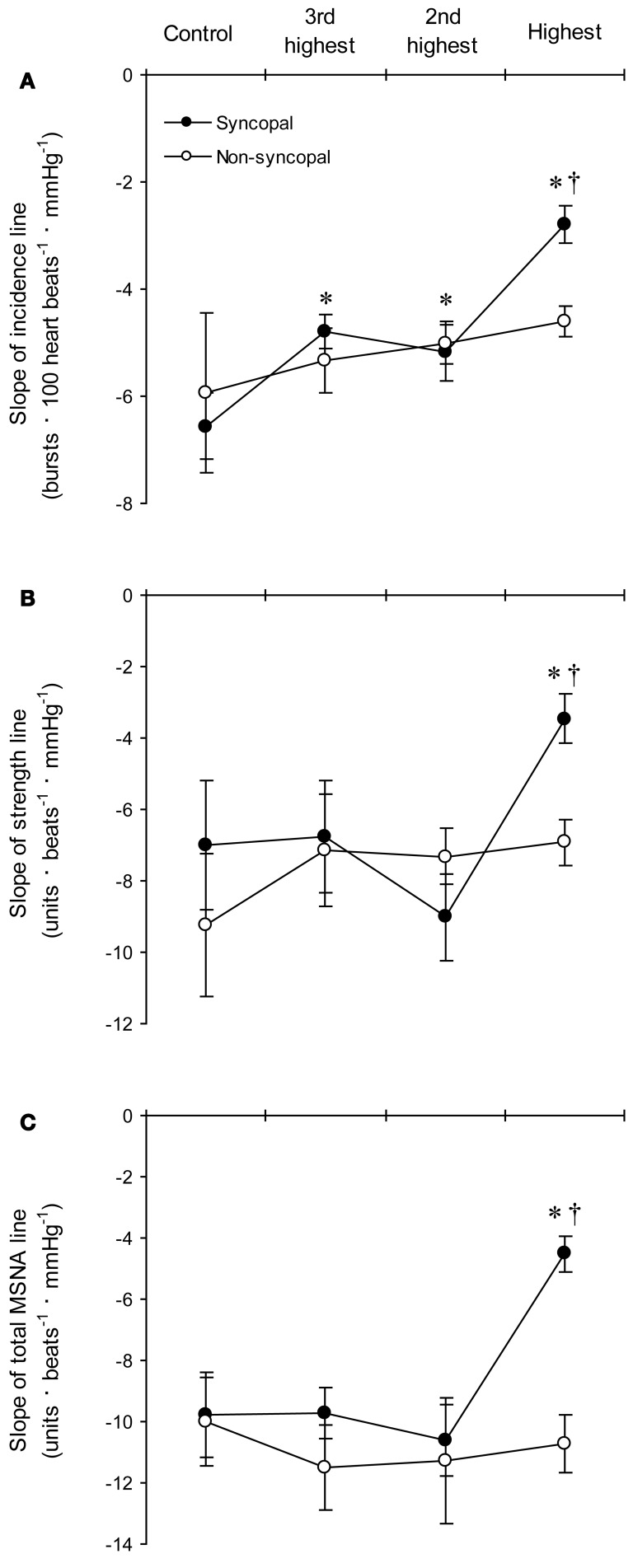
**The group averaged values of the slopes of the linear regression lines relating DAP to burst incidence (A), burst strength (B), and total MSNA (C) in the syncopal (*n* = 9 subjects) and non-syncopal (*n* = 3 subjects) subjects in the control situation and at the three highest LBNP levels**. ^*^P < 0.05 vs. control. ^†^*P* < 0.05 vs. the 2nd LBNP level. (Reproduced with permission from Ichinose et al., 2006).

We used the abovementioned technique to examine the ABR-mediated beat-to-beat control of MSNA during supine rest (control) and progressive, stepwise increases in LBNP, which was incremented by −10 mmHg every 5 min until presyncope or −60 mmHg was reached (Ichinose et al., 2006). Figure 4 shows raw recordings of arterial blood pressure and MSNA during supine rest (control) and at LBNPs of −50 mmHg and −60 mmHg for a subject who experienced symptoms of presyncope at LBNP = −60 mmHg. Figure 5 shows the linear regression lines relating burst incidence, burst strength, and total MSNA to DAP for a non-syncopal subject (**A**,**C**,**E**) and for a syncopal subject who experienced syncope at LBNP = −60 mmHg (**B**,**D**,**F**). Finally, Figure 6 shows the group averaged values of the slopes of the linear regression lines relating burst incidence (A), burst strength (B), and total MSNA (C) to DAP in syncopal and non-syncopal subjects during the control period and at the three highest LBNP levels. The points corresponding to the average DAP on the regression lines relating burst incidence or total MSNA to DAP were taken as the prevailing points for a given relationship and as an index of the MSNA corresponding to the ABR operating pressure. For all of the subjects, there was a significant negative correlation between burst incidence and DAP and total MSNA and DAP under all conditions. Not all of the subjects showed a significant negative correlation between burst strength and DAP, however. And even among those who did show a significant relationship, the correlation coefficient was smaller than those relating burst incidence or total MSNA to DAP. Because of the weakness of the relationship, we did not calculate the prevailing point for the regression line relating burst strength to DAP.

With increasing LBNP, the linear relationship between burst incidence and DAP and the prevailing point were both gradually shifted upward until LBNP = −30 mmHg (Figures 5A,B). LBNPs of −40 mmHg and above elicited no further upward shift in the prevailing point from that seen at −30 mmHg. In addition, the linear relationship between burst strength or total MSNA and DAP and the prevailing points were all shifted progressively upward with increasing LBNP until −60 mmHg (Figures 5C–F). Although the slope of the relationships remained constant at all LBNPs tested, except at the level where syncope occurred, the slope of the relationship between DAP and burst incidence was reduced at LBNPs of −40 mmHg and higher (vs. control). In syncopal subjects, comparisons among the slopes obtained at LBNPs where syncope occurred, at the two preceding LBNP levels, and during the control period revealed that the slopes of the burst incidence, burst strength, and total MSNA lines were all significantly less negative at LBNPs where syncope occurred than during the control period or the preceding LBNP levels (Figure 6). By contrast, the slopes did not significantly differ among the highest three LBNP levels in non-syncopal subjects.

### Possible mechanisms for the modulation of ABR-mediated beat-to-beat control of MSNA under orthostatic stress

It has been suggested that the upward resetting of ABR control in response to orthostatic stress facilitates the activation of sympathetic nerve activity, thereby contributing to the prevention of postural hypotension (Ichinose et al., 2004b; Kamiya et al., 2005b). Our results suggest that in humans, MSNA progressively increases in response to increasing orthostatic stress through the gradual upward resetting of ABR control over MSNA. Given that ABR control over total MSNA is dependent on its control over both burst incidence and strength (Ichinose et al., 2004b,c), the upward resetting of ABR control over total MSNA elicited by LBNPs up to −30 mmHg would reflect the upward resetting of ABR control over both burst incidence and strength. On the other hand, because there is no further upward resetting of ABR control of burst incidence at LBNPs higher than −30 mmHg, the upward resetting of ABR control over burst strength would be the major cause of the further upward resetting of ABR control over total MSNA seen at LBNPs above −30 mmHg. These results suggest the mechanisms underlying the progressive upward resetting of ABR control over MSNA in response to increasing orthostatic stress are not uniform over the range of mild to very severe orthostatic stress.

We cannot provide a definitive explanation of the mechanisms responsible for the progressive modulation of ABR-mediated beat-to-beat control over MSNA that occurs in response to increasing orthostatic stress. It is possible this modulation reflects the interactions between ABR and other systems contributing to the regulation of sympathetic nerve activity. For example, as already mentioned, afferent signaling from the cardiopulmonary baroreceptors may influence the ABR at integration sites within the CNS (Victor and Mark, 1985; Pawelczyk and Raven, 1989; Ogoh et al., 2003; Charkoudian et al., 2004, 2005; Ichinose et al., 2004a), such as the NTS (Spyer, 1990; Li et al., 1998; Potts et al., 2003). In particular, a progressive modulation of the ABR control over the incidence and strength of sympathetic bursts during gradual unloading of the cardiopulmonary baroreceptors, as described above, could only be affected within such a site of integration.

Alternatively, the modulation of ABR control over MSNA might be associated with the unloading of the arterial baroreceptors themselves. For example, the upward shift of the DAP-MSNA relationship during LBNP could reflect increased MSNA due to unloading of the arterial baroreceptors induced by reductions in systolic arterial pressure and pulse pressure, even though the average DAP remained unchanged. Moreover, the DAP-MSNA relationship could approach the upper plateau of the sigmoidal baroreflex function through unloading of the arterial baroreceptors; if so, this could be one cause for the reduction in the slope of the DAP-burst incidence line at high levels of LBNP and the reduction in the slope of the DAP-MSNA (all three parameters) line prior to the orthostatic syncope. Earlier studies suggested that LBNP levels too low to cause hypotension (less than −20 mmHg) selectively unload cardiopulmonary baroreceptors without changing afferent activity from arterial baroreceptors (Zoller et al., 1972; Johnson et al., 1974; Rowell, 1986). If so, the modulation of ABR control over MSNA under mild LBNP ought to be induced by unloading the cardiopulmonary baroreceptors without unloading arterial baroreceptors. It has been shown, however, that the dimensions of the aortic baroreceptive areas are reduced during mild LBNP (Taylor et al., 1995). In light of that finding, the modulation of the beat-to-beat control over MSNA mediated by ABR during progressive increases in LBNP may be associated with unloading of both the cardiopulmonary and arterial baroreceptors. In any case, the precise mechanisms remain to be elucidated.

### ABR function during development of orthostatic syncope

The function of the ABR during development of orthostatic syncope is largely unknown. Ogoh et al. (2004) reported that as presyncopal symptoms develop during head-up tilt in humans, the sensitivity of the carotid baroreflex control over HR and MAP declines substantially and then declines still further during syncope. In addition, Kamiya et al. (2005a) reported that low frequency oscillations in MSNA are reduced though MSNA remains elevated during the early phase of development of head-up tilt-induced syncope. These results suggest that one or more control systems governing MSNA are modulated prior to the inhibition of MSNA during the development of orthostatic syncope. Our results confirm those earlier reports and show that the sensitivities of the ABR control over burst incidence, burst strength, and total MSNA are all substantially diminished prior to the apparent inhibition of MSNA during the development of orthostatic syncope. We believe it is unlikely that this reflects a sudden reduction in the responsiveness of the arterial baroreceptors; more likely, the influence of the arterial baroreceptors on MSNA is inhibited within the CNS. It has been hypothesized that the withdrawal of sympathetic nerve activity and the concomitant bradycardia seen during orthostatic syncope (vasovagal reaction) is initiated from receptors situated in the inferior part of the left ventricle, which are activated by the combination of low cardiac filling secondary to venous pooling and an increase in the inotropic state of the heart (often termed the “Bezold-Jarisch reflex”) (Madsen and Secher, 1999; Kinsella and Tuckey, 2001; Campagna and Carter, 2003). We suggest that during the development of orthostatic syncope, the cardiac depressor reflex may be activated and may initially act within the CNS to inhibit the effect of ABR on sympathetic nerve activity. Subsequently, ABR function may be overridden completely, so that withdrawal of sympathetic activity and bradycardia occur concomitantly with the severe hypotension that leads to syncope. In contrast to the results of our study and abovementioned studies, Vaddadi et al. (2010) showed that in most patients with vasovagal syncope, the syncope occurs despite MSNA being maintained. Similarly, Fu et al. (2012) reported that MSNA withdrawal is not always seen in healthy subjects who develop presyncope during head-up tilt. They suggested that intrinsic impairment of vasomotor responsiveness and sympathetic baroreflex function is not the cause of neurally mediated presyncope. Although the reason for the apparent discrepancy is not clear, the orthostatic syncope could have been induced via several paths, including a marked fall in cardiac output, a reduced vasomotor response, vasodilator mechanisms, and withdrawal of sympathetic activity. Further studies will be needed before we can fully explain the role of ABR in the development of orthostatic syncope.

## Summary

In summary, dynamic carotid baroreflex-induced responses are modulated under orthostatic stress, as exemplified by the augmentation of the MSNA, MAP, and HR responses to carotid baroreflex unloading, and the shorter suppression period of MSNA, comparable reduction and faster recovery of MAP, and greater HR response to carotid baroreflex stimulation. In addition, ABR-mediated beat-to-beat control over burst incidence, burst strength, and total MSNA are all progressively modulated as orthostatic stress is increased until induction of syncope, and the sensitivity of ABR control over the aforementioned MSNA variables is substantially reduced during the development of syncope. We suggest that in humans, the modulation of ABR function under orthostatic stress may be one of the mechanisms maintaining blood pressure and limiting orthostatic hypotension, and impairment of ABR control over sympathetic vasomotor activity leads to the severe hypotension associated with orthostatic syncope.

### Conflict of interest statement

The authors declare that the research was conducted in the absence of any commercial or financial relationships that could be construed as a potential conflict of interest.
